# 

**Published:** 1903-01-17

**Authors:** 


					THE hospital. "The standard of highest purity."?Lancet. Jan. 17th, 1903.
CADBURY'S ww CADBURY'S
COO OA IM I* COOOA
ABSOLUTELY PURE. jgh <S? NO DRUGS OR ALKALIES USED.
No. K51. Vol. XXXIII. [aNewSerl] SATURDAY,
Jan. 17 th, 1903.
[Sabsariptionjerma,] pRICH TWOPENCE.

				

